# Surveillance and outcomes after curative resection for gastroesophageal adenocarcinoma

**DOI:** 10.1002/cam4.2948

**Published:** 2020-03-04

**Authors:** Di M. Jiang, Chihiro Suzuki, Osvaldo Espin‐Garcia, Charles H. Lim, Lucy X. Ma, Peiran Sun, Hao‐Wen Sim, Akina Natori, Bryan A. Chan, Stephanie Moignard, Eric X. Chen, Geoffrey Liu, Carol J. Swallow, Gail E. Darling, Rebecca Wong, Raymond W. Jang, Elena Elimova

**Affiliations:** ^1^ Department of Medical Oncology Princess Margaret Cancer Centre University Health Network University of Toronto Toronto Ontario Canada; ^2^ Department of Biostatistics Princess Margaret Cancer Centre University Health Network University of Toronto Toronto Ontario Canada; ^3^ Department of Surgical Oncology Mount Sinai Hospital Princess Margaret Cancer Centre University Health Network University of Toronto Toronto Ontario Canada; ^4^ Division of Thoracic Surgery Department of Surgery Toronto General Hospital University Health Network University of Toronto Toronto Ontario Canada; ^5^ Radiation Medicine Program Princess Margaret Cancer Centre Ontario Cancer Institute University Health Network University of Toronto Toronto Ontario Canada

**Keywords:** gastric cancer, recurrence, surveillance, survival

## Abstract

**Background:**

The goal of surveillance testing is to enable curative salvage therapy through early disease detection, however supporting evidence in gastroesophageal adenocarcinoma is limited. We evaluated frequency of successful salvage therapy and outcomes in patients who underwent surveillance.

**Methods:**

A single‐site, retrospective cohort study was conducted to identify all patients who received curative resection for gastroesophageal adenocarcinoma. Surveillance testing were those investigations not triggered by abnormal symptoms, physical examination, or blood tests. Successful salvage therapy was any potentially curative therapy for disease recurrence which resulted in postrecurrence disease‐free survival ≥2 years. Time‐to‐event data were analyzed using the Kaplan‐Meier method and log rank tests.

**Results:**

Between 2011 and 2016, 210 consecutive patients were reviewed. Esophageal (14%), gastroesophageal junction (40%), and gastric adenocarcinomas (45%) were treated with surgery alone (29%) or multimodality therapy (71%). Adjuvant therapy was administered in 35%. At median follow‐up of 38.3 months, 5‐year overall survival (OS) rate was 56%. Among 97 recurrences, 53% were surveillance‐detected, and 46% were symptomatic. None was detected by surveillance endoscopy. Median time‐to‐recurrence (TTR) was 14.8 months. Recurrences included locoregional only (4%), distant (86%), and both (10%). Salvage therapy was attempted in 15 patients, 4 were successful. Compared to symptomatic recurrences, patients with surveillance‐detected recurrences had longer median OS (36.2 vs 23.7 months, *P* = .004) and postrecurrence survival (PRS, 16.5 vs 4.6 months, *P* < .001), but similar TTR (16.2 vs 13.3 months, *P* = .40) and duration of palliative chemotherapy (3.9 vs 3.3 months, *P* = .64).

**Conclusions:**

Among patients surveyed, 96% of recurrences were distant, and salvage therapy was successful in only 1.9% of patients. Longer OS in patients with surveillance‐detected compared to symptomatic recurrences was not associated with significant earlier disease detection, and may be contributed by differences in disease biology. Further prospective data are warranted to establish the benefit of surveillance testing in gastroesophageal adenocarcinoma.

## Graphical abstract

For resected gastroesophageal adenocarcinoma treated at our institution, routine surveillance testing rarely enabled successful salvage therapy, did not detect recurrence earlier, and did not extend duration of palliative chemotherapy. Further prospected data is warranted to establish the role of surveillance testing in gastroesophageal adenocarcinoma.

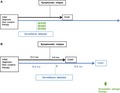

## 1 INTRODUCTION

Despite advances in curative therapy, recurrence rates of gastroesophageal adenocarcinoma remain high at approximately 40%‐50%.^1^, ^2^, ^3^ Because of the poor outcomes of recurrent disease, earlier detection at the asymptomatic stage may allow timely intervention and improved outcomes. However, routine surveillance testing in patients with resected locally advanced gastroesophageal adenocarcinoma is not yet supported by robust supprting evidence. Randomized controlled trials evaluating the utility of routine surveillance are lacking. Although previous retrospective studies have been attempted, important limitations including sample size, outdated preoperative staging, potentially suboptimal curative therapy, and surveillance imaging techniques^4^, ^5^, ^6^ limit the interpretation of these data. Other studies conducted in Asia also have limited generalizability to Western populations given their distinct disease characteristics and treatment strategies.^7^ Current guidelines from major international cancer organizations such as National Comprehensive Cancer Network (NCCN), American Society of Clinical Oncology (ASCO), European Society of Medical Oncology (ESMO), and Cancer Care Ontario (CCO) as well as international expert panels^8^ have yet to specifically define the optimal surveillance strategy. As a result, surveillance protocols vary widely among institutions and physicians.^2^

In this study, we examined the benefit of routine surveillance testing following curative resection of gastroesophageal adenocarcinoma. We aimed to evaluate (a) recurrence patterns, (b) frequency of successful salvage therapy, and (c) outcomes for patients with asymptomatic recurrence detected by surveillance testing compared to those with symptomatic recurrence.

## 2 MATERIALS AND METHODS

### 2.1 Patient identification and data collection

From the institutional Registry of Princess Margaret Cancer Centre (PMCC), we identified consecutive patients with esophageal, gastroesophageal junction (GEJ), and gastric adenocarcinoma who had curative resection between 2011 and 2016 and subsequent surveillance (Supplementary 1). This study was approved by the University Health Network institutional review board. Prior to data collection from electronic medical record, all extractors underwent data dictionary training. All data were verified by a second investigator (DMJ) for quality assurance. Any discrepancies were discussed with a third investigator (EE) to reach consensus.

### 2.2 Primary curative therapy

After initial diagnosis, all patients had standard clinical staging investigations including esophagogastroduodenoscopy (EGD) ± endoscopic ultrasound, and computerized tomography (CT) scan of the chest/abdomen/pelvis. Positron emission tomography for esophageal and GEJ cancers and diagnostic laparoscopy for gastric cancers were also used for staging. All patients had clinical and pathological TNM staging according to the 2002 American Joint Committee on Cancer (AJCC) system 7th edition. Curative treatment options were discussed in a gastroesophageal cancer‐specific multidisciplinary tumor board with input from experienced radiologists, surgical oncologists, radiation oncologists, medical oncologists, and pathologists whenever possible.

Neoadjuvant chemoradiation (according to the CROSS trial for patients treated after 2012) were used to treat esophageal, Siewert I, and selected Siewert II GEJ tumors.^9^ Other Siewert II GEJ tumors were treated either with neoadjuvant chemoradiation or perioperative chemotherapy. Perioperative chemotherapy (MAGIC^10^ prior to, and FLOT regimen^11^ after June 2017) was used for gastric and Siewert III GEJ tumors. Adjuvant chemoradiation based on the MacDonald protocol^12^ or adjuvant chemotherapy were recommended for fit patients treated with upfront resection.

### 2.3 Surveillance testing

Surveillance investigations including bloodwork, imaging, or EGDs were performed at the discretion of treating physicians. There were no uniform institutional recommendations regarding the optimal surveillance modality, frequency and duration due to the lack of high level evidence in the literature. All investigations conducted following curative therapy were reviewed in detail with respect to their indications. Surveillance tests were those performed in the absence of suspected disease recurrence, such as abnormal symptoms, physical examination, or blood tests, and were usually ordered months in advance. Followup tests were performed to assess equivocal findings on surveillance tests (such as indeterminate pulmonary nodule), sooner than the usual surveillance interval. Confirmatory tests were usually performed within a few weeks, triggered by abnormal symptoms, physical examination or blood tests. Method of recurrence detection was classified as surveillance‐detected (surveillance or followup testing), or symptoms‐detected. Intensive (surveillance interval of ≤4 months with respect to imaging at any time during the surveillance period) versus nonintensive surveillance strategy (surveillance imaging interval >4 months) were also assessed. The interval of 4 months was chosen based on published expert recommendations, and similar definitions used in trials of colorectal cancer.^16^, ^17^ The Italian Research Group for Gastric cancer defined CT scans every 6 months as the intensive follow‐up protocol in gastric cancer.^18^

### 2.4 Recurrence patterns and salvage therapy

Upon suspicion of first recurrence (either by surveillance investigations or symptoms), complete re‐staging with CT chest/abdomen/pelvis was performed for further evaluation. Patients underwent further workup specific if indicated. Histological confirmation of recurrence was obtained at the discretion of the treating physician. Equivocal cases were discussed in our gastroesophageal cancer‐specific multidisciplinary tumor board. Diagnosis of recurrence was adjudicated by pathologic confirmation or by findings on investigations that led to changes in management.

Recurrence patterns were characterized as locoregional (LRR) or distant recurrence (DR) or both. LRR occurred near the surgical anastomosis, within the gastroesophageal lumen, or regional lymph nodes. DR was any recurrence beyond locoregional, and was subcategorized as nonvisceral (bone, peritoneum, or both), visceral (any other site including distant lymph nodes), or both nonvisceral and visceral.

Possibilities of salvage therapy were discussed within a multidisciplinary team whenever possible. Attempted salvage therapy was any intervention for recurrent disease with a curative intent. Successful salvage therapy resulted in post‐recurrence disease‐free survival (DFS) of ≥2 years. This interval was chosen arbitrarily, taking into consideration the median survival of patients with relapsed metastatic gastric cancer is often less than 12 months at experienced centers,^13^, ^14^ and the same definition has been used in the literature.^15^

### 2.5 Statistical analysis

Descriptive statistics were used where appropriate. Time‐to‐event data were analyzed using the Kaplan‐Meier curves and compared using log rank tests. Time‐to‐recurrence (TTR), DFS, and overall survival (OS) were calculated from date of initial diagnostic biopsy to account for timelines of various perioperative modalities. Postrecurrence survival (PRS) was calculated from date of initial recurrence detection. Patients with incomplete follow‐up at the time of study analysis were censored at the time of last follow‐up. Duration of surveillance testing was calculated from completion of curative therapy to last surveillance test performed. For patients who received salvage therapy, surveillance resumed at the first follow‐up visit following completion of salvage therapy.

Outcomes of patients with surveillance‐detected and nonsurveillance‐detected recurrences were compared using a predefined multivariable Cox proportional hazards regression model, adjusting for age, sex, Siewert class, and high risk pathologic staging (T3/T4 or node positive). A more comprehensive multivariable model including Charlson comorbidity index, marital status, modalities of primary treatment received, and other potential confounders could not be employed given the limited sample size and risk of overfitting.

All statistical analyses were performed using R version 3.1.2 (R Core Team 2014). All *P* values were two‐sided and <0.05 was considered statistically significant. Missing data were dealt with using the listwise deletion method.

## 3 RESULTS

### 3.1 Patient characteristics

Between 2011 and 2016, 210 consecutive patients with resected esophageal (14%), GEJ (40%), and gastric adenocarcinoma (45%) who underwent surveillance were identified. Patients were predominantly male (73%), non‐Asian (81%), with a median age of 64.1 years (range 28.9‐86.2). Overall, 68% of patients received their curative resection at PMCC or Mount Sinai Hospital. Median number of lymph nodes resected was 25 (range 6‐88). R0 resection rate was 91%. Majority had high risk (T3/T4 or node positive) pathological staging (72%). Patients received surgery alone (29%), perioperative chemotherapy (26%), or perioperative chemoradiation (45%) (Table 1).

**Table 1:** Baseline characteristics

Baseline characteristics	n (%)
Median age (years)	64.1 (range 28.9, 86.2)
Male	153 (73)
Asian	39 (19)
ECOG	
0	90 (43)
1	111 (53)
≥2	9 (4)
Smoking	
Current	37 (18)
Never	86 (41)
Ex‐smoker	81 (39)
Unknown	6 (2)
Alcohol	
Never	65 (31)
Occasional	78 (37)
Frequent	35 (17)
Past	19 (9)
Unknown	13 (6)
Past malignancy	33 (16)
Adenocarcinoma subtype	
Pure adenocarcinoma	154 (74)
Mucinous	8 (4)
Signet ring	43 (20)
Neuroendocrine	0
Unknown	3 (1)
Grade	
G1	22 (11)
G2	61 (29)
G3	101 (48)
Unknown	26 (12)
HER2 positive	34 (16)
Siewert	
Esophagus	29 (14)
Gastroesophageal junction	86 (41)
AEG 1	35 (17)
AEG 2	35 (17)
AEG 3	15 (7)
Gastric	95 (45)
Clinical staging	
I	27 (13)
II	86 (41)
III	97 (46)
Primary treatment modality	
Surgery alone	60 (29)
Surgery plus adjunctive therapy	150 (71)
Neoadjuvant chemotherapy only	19 (9)
Adjuvant chemotherapy only	11 (5)
Neoadjuvant and adjuvant chemotherapy	25 (12)
Neoadjuvant chemoradiation only	56 (27)
Adjuvant chemoradiation only	29 (14)
Neoadjuvant chemotherapy and adjuvant chemoradiation	7 (3)
Neoadjuvant chemoradiation and adjuvant chemotherapy	3 (1)
Surgery type	
Esophagectomy	25 (12)
Subtotal gastrectomy	75 (36)
Total gastrectomy	24 (11)
Esophagogastrectomy	84 (40)
Unknown	2 (1)
Number of lymph nodes resected	Median 25 (range 6, 88)
6‐10	8 (4)
11‐20	56 (27)
21‐30	69 (33)
31‐40	43 (20)
41‐50	17 (8)
>50	13 (6)
Missing	4 (2)
Margin	
Free (R0)	192 (91)
Positive margin (R1)	13 (6)
Proximal	5 (2)
Distal	1 (0)
Radial	7 (3)
Unknown	5 (2)
Pathologic staging TNM	
Tx/Tis/T0N0	15 (7)
T1N0	27 (13)
Tx‐T1N1‐3	17 (8)
T2N0	16 (8)
T2N1‐3	13 (6)
T3N0	25 (12)
T3N1	19 (9)
T3N2	23 (11)
T3N3	28 (13)
T4N0	12 (6)
T4N1, T4N2, T4N3	15 (7)
Pathologic staging	
0	15 (7)
I	35 (17)
II	47 (22)
III	113 (54)
High risk path staging (T3/T4 or N positive)	152 (72)
T3/T4	123 (59)
N positive	114 (54)

Abbreviations: AEG, adenocarcinoma of the esophagogastric junction; ECOG, The Eastern Cooperative Oncology Group (ECOG) performance status; N positive, lymph node positive.

### 3.2 Surveillance patterns, recurrence patterns, method of detection

Surveillance protocols varied (Supplementary 2). Most common surveillance tests were imaging (90%), either alone or combined with tumor markers and EGDs. Imaging modalities consisted of mostly CT scans including chest abdomen and pelvis (97%). Among 41 patients (20%) who received surveillance EGD, 86% had subtotal gastrectomy. Tumor markers were rarely utilized (7%). Among patients who had clinical visits alone (9%), most recurred within the first 6 months before surveillance investigations took place. Median duration of surveillance in patients free of recurrence was 34.7 months (95% CI: 0‐82.4).

Among the 97 recurrences (46%), 96% included DR. Few patients had LRR alone (n = 4, 4%), all of which had R0 resection with >20 lymph nodes resected. Disease recurrence was uncommon beyond 3 years (9 out of 97 patients). Recurrences were detected by surveillance testing in 51 patients (53%) and symptoms in 45 (46%). There was one patient whose method of recurrence detection was unavailable. Surveillance CT detected 50 recurrences, EGD detected none. Only 1 recurrence was detected by a tumor marker (CA‐125 as part of a clinical trial).

Overall, follow‐up testing ordered to assess equivocal findings on surveillance testing were performed in 90 patients (43%), and confirmed recurrence in 35 (39%). Second malignancy was documented in 6 patients found during surveillance, mostly metastatic and unlikely to be treatment related (Supplementary 3).

### 3.3 Survival outcomes

**Figure 1:**
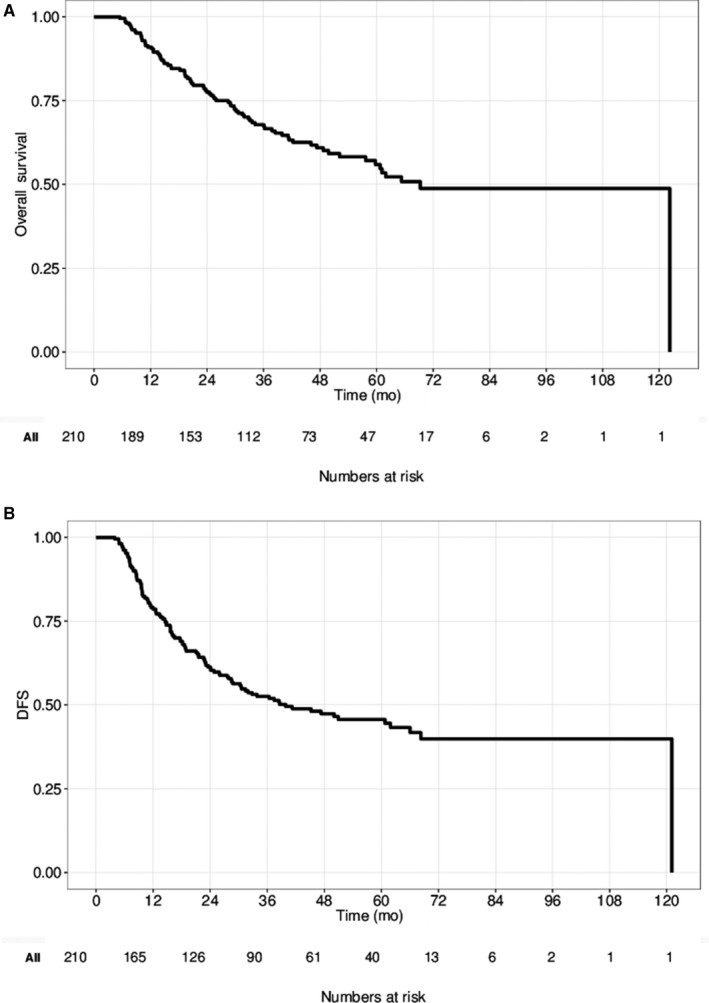
A, Overall survival (OS), (B) Disease‐free survival (DFS) of the overall study cohort

After a median follow‐up of 38.3 months (range 5.5‐122.3), 3‐ and 5‐year OS rates were 68% (95% CI: 62%‐75%) and 56% (95% CI: 49%‐64%), respectively (Figure 1A). There were 85 deaths (40%) during the follow‐up period. DFS rates at 3 and 5 years were 53% (95% CI 46%‐60%) and 46% (95% CI: 39%‐53%), respectively (Figure 1B). For those who recurred, median TTR and PRS were 14.8 months (95% CI: 11.6‐18.1) and 10.7 months (95% CI: 6.6‐16.2), respectively.

Recurrence associated with symptoms, nongastric primaries, high risk pathologic staging, and non‐Asian ethnicity were associated with inferior OS. On multivariable analysis, symptomatic recurrence (HR 2.46, 95% CI: 1.45‐4.16, *P* < .001) and high risk pathologic staging (HR 2.29, 95% CI: 1.04‐5.03, *P* = .039) remain associated with poorer OS. Symptomatic recurrence was associated with poorer PRS (HR 2.79, 95% CI: 1.68‐4.65, *P* < .001), but not DFS and TTR (*P* = .11).

**Figure 2:**
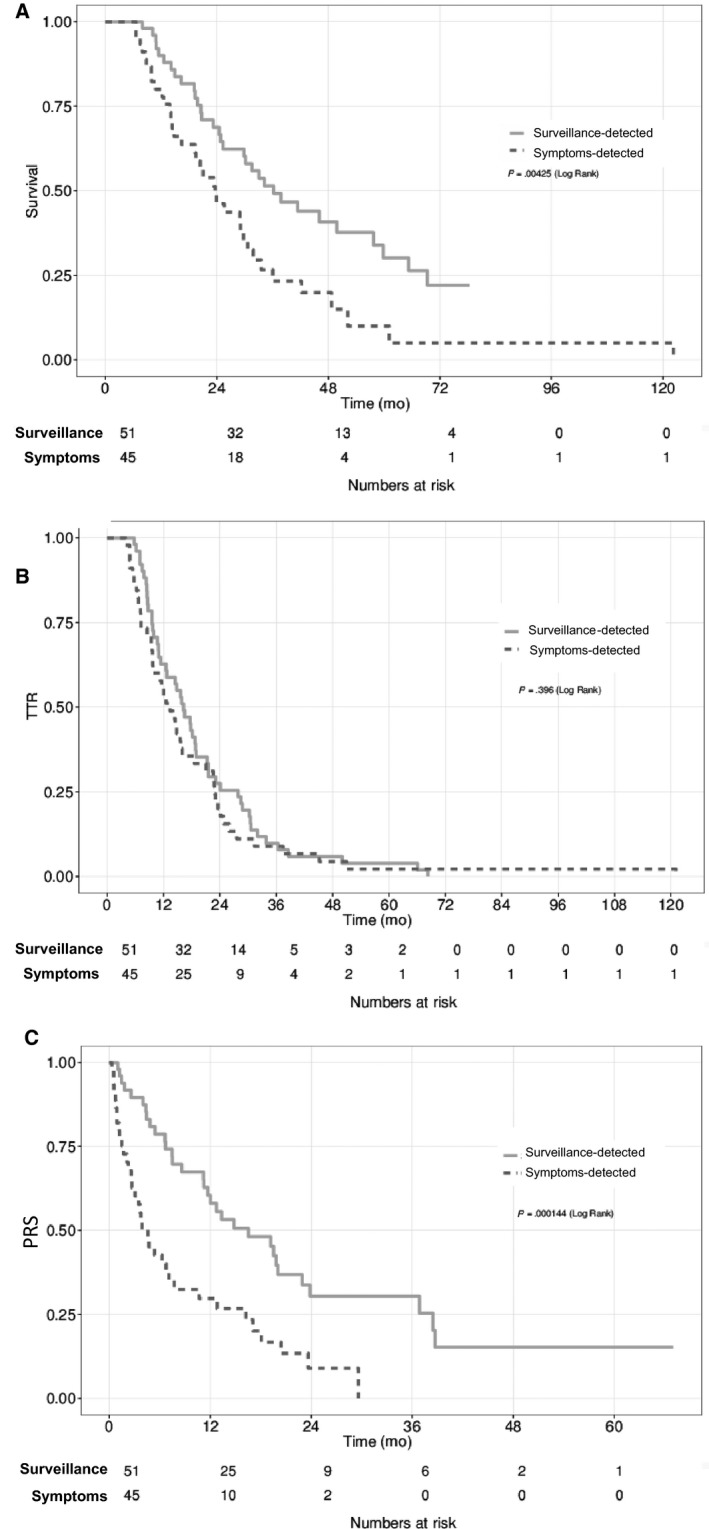
A, Overall survival (OS), (B) Time‐to‐recurrence (TTR), (C) postrecurrence survival (PRS) in patients with recurrences detected by surveillance and symptoms

Patients with surveillance‐detected recurrences had similar proportions of high grade tumors, signet ring histology, positive margins, high‐risk pathologic staging, and perioperative treatment modalities compared to those with symptomatic recurrences. However, on multivariable analysis, the former group had longer median PRS (16.5 vs 4.6 months, HR 2.79, 95% CI: 1.68‐4.65, *P* < .001) and OS (36.2 vs 23.7 months, HR 2.46, 95% CI: 1.45‐4.16, *P* < .001) (Figure 2A,C). TTR was similar (16.2 vs 13.3 months, *P* = .40) (Figure 2B). Patients who underwent intensive surveillance imaging had worse disease features and outcomes than those who underwent nonintensive surveillance imaging (Supplementary 4).

### 3.4 Treatment postrecurrence

Attempted salvage therapy was performed in 16 patients (8%), 4 (2%) were successful (Supplementary 5 and 6), 6 (3%) were unsuccessful, and 6 (3%) had immature follow‐up (Table A.6). Among patients who recurred, 46 (47%) received palliative systemic therapy—28 had surveillance‐detected and 18 had symptomatic recurrence. Median duration of palliative chemotherapy was 3.9 and 3.3 months, respectively (Supplementary 5).

## 4 DISCUSSION

**Figure 3:**
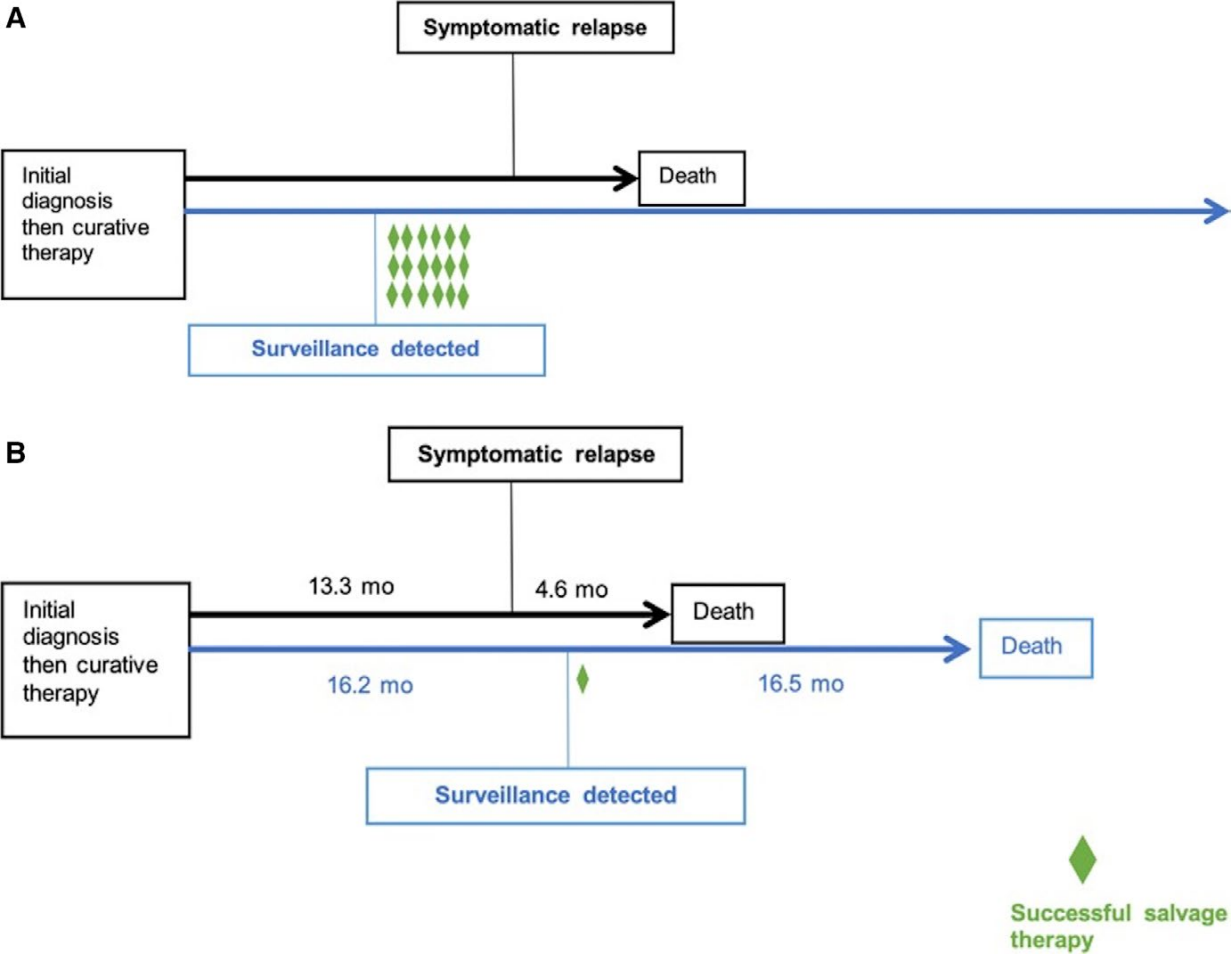
A, Theoretical outcomes of patients who develop recurrence if surveillance testing enables earlier disease detection, higher success rate of salvage therapy or prolonged duration of palliative systemic therapy. B, Actual outcomes of patients who underwent surveillance

The main goal of surveillance testing is early detection of asymptomatic recurrence, enabling successful curative salvage therapy to improve OS and quality of life (Figure 3A). In colorectal cancer, randomized clinical trials have demonstrated a modest survival advantage with routine surveillance imaging, tumor markers, and colonoscopy.^19^ Selected patients with liver and/or lung metastases can be salvaged with metastasectomy plus chemotherapy with cure rates up to 40%, which provides the rationale for surveillance.^20^ In gastroesophageal adenocarcinoma, salvage therapy such as re‐resection, metastasectomy, or chemoradiation is rarely possible or successful,^21^ thus the rationale for surveillance is lacking. While routine followup is an integral part of patient care to minimize treatment sequelae and promote overall health, our data suggest routine surveillance testing may not detect recurrences earlier to enable curative intervention in all patients (Figure 3B). Surveillance testing likely was beneficial for the rare patients who received successful salvage therapy in our study, however for the majority of patients who recurred, routine surveillance testing did not detect recurrences amenable for salvage therapy. There were 10 more patients in the surveillance‐detected group who received palliative systemic therapy, however the median duration of treatment was similar compared to the symptomatic group. Conceivably, as systemic therapy cannot improve quality of life for asymptomatic disease, immediate treatment initiation may not be warranted. These results suggest that many of these patients did not benefit from routine surveillance testing with respect to early recurrence detection, and their follow‐up were likely not cost‐effective strategies. This highlights the need for a validated individualized, risk‐tailored approach to enhance the utility and feasibility of surveillance testing in this disease.

In patients with surveillance‐detected recurrences, both PRS and OS were approximately 12 months longer than patients with symptomatic recurrences. Others have shown strikingly similar results.^1^, ^3^ The favorable outcomes in the former group cannot be explained by earlier recurrence detection by surveillance testing (i.e., lead time bias) alone. Patients with surveillance‐detected recurrences had TTR almost 3 months longer than patients with symptomatic recurrences, although this did not meet statistical significance probably due to limited sample size. TTR is a strong prognostic factor PRS and OS,^3^, ^13^, ^22^ and is likely contributed by disease biology—asymptomatic recurrences are inherently more slow‐growing than symptomatic recurrences, and are more likely to be detected by interval surveillance tests (i.e., length time bias). Consistent with this theory, our multivariable analysis model demonstrated that asymptomatic recurrence was a strong prognostic factor for superior PRS and OS. Other studies have also reported similar associations across different surveillance strategies.^2^, ^23^ Given the above data, the true magnitude of impact of surveillance testing on outcomes in patients with surveillance‐detected recurrences cannot be ascertained from biological variables which also dictate outcomes. It is possible that some patients with asymptomatic disease and indolent biology may have benefitted from surveillance testing, however this requires confirmation from future randomized trials. Unfortunately, randomized controlled trials evaluating the role of surveillance is unlikely to be achieved in the near future.^8^

Four large series attempted to characterize the benefit of surveillance testing in Western patients with gastroesophageal adenocarcinoma,^1^, ^2^, ^15^, ^18^ using older cohorts from the periods of 2001‐2010,^1^, ^2^ 1998‐2009,^17^ and 1995‐2014.^13^ Perioperative treatment for many gastric cancer patients were likely suboptimal prior to the publication of the pivotal MAGIC trial in 2006.^13^, ^17^ Similarly, prior to the CROSS trial published in 2012, use of neoadjuvant chemoradiation was likely limited for patients with esophageal cancer. In two cohorts, most patients with gastric or GEJ tumors received neoadjuvant chemoradiation, which is not the standard of care.^2^, ^15^ Our study adds updated and comprehensive data following modern standard of care multimodality perioperative therapy. It also allows a broad outlook on surveillance testing including followup testing, postrecurrence treatments and second malignancies discovered during surveillance which were previously unpublished.

Higher LRR rates up to 24% have been reported.^24^ However, in the previous study, 5‐year OS rate was poor at 39.3%, median number of lymph nodes resected was lower (17) and 59% of patients received D2 resection. Extent of node dissection was significantly associated with LRR, suggesting that suboptimal resection was an important factor. LRR rates in the landmark MAGIC and CROSS trials were 14% and 3%, respectively, and only 40% of patients received D2 dissection in the MAGIC trial.^10^, ^25^ Our data demonstrate that following aggressive node dissection, LRR alone is rare.

Surveillance tests varied among physicians as the optimal surveillance strategy is unknown. Most physicians used imaging (90%) for surveillance, 97% of which were CT scans. While CTs detected most recurrences, routine EGDs were not useful. Surveillance endoscopies were not commonly used given lack of supporting evidence and true isolated local recurrence is uncommon.^8^ Tumor markers were rarely used given lack of evidence. The incremental value of surveillance tumor markers in addition to cross‐sectional imaging remains undefined.^8^, ^18^ A prospective trial has shown tumor markers do not detect gastric cancer recurrence earlier than imaging.^26^ As previously shown, surveillance testing may provide the highest yield within the first 3 years.^1^, ^3^ Frequency of surveillance testing does not seem to confer benefit. However, patients with more aggressive disease are likely preferentially selected for intensive surveillance, which results in confounding by indication. This is supported by the greater proportions of patients with high risk pathologic staging, positive margins, and proximal primaries in the intensive surveillance group. Whether intensive surveillance improves patient outcomes in this disease can only be answered in a comparative prospective clinical trial.

The potential benefit of routine surveillance testing needs to be considered with its potential harms, and informative discussions with patients are critical. While there is no clear association between cumulative diagnostic radiation exposure and secondary malignancies in adults, the average radiation dose for a CT chest/abdomen/pelvis with intravenous contrast is comparable to 9 years of background radiation,^27^ which may be concerning for many patients. Almost half of the patients underwent additional follow‐up testing including further imaging and invasive biopsies, with the majority proving false positivity of initial surveillance tests, which likely added unnecessary risks, patient anxiety^28^, and cost.^29^ Substantial financial implications also exist,^15^ and Choosing Wisely campaign recommends avoiding surveillance testing if it is not expected to improve survival or quality of life.^30^

Inherent to the retrospective nature of this study, several limitations exist. The exact role of routine surveillance in patients with resected gastroesophageal adenocarcinoma and how to best select patients for appropriate surveillance remains to be answered in prospective clinical trials. The outcomes reported in this study (recurrence patterns and survival outcomes) pertain only to those patients who received any routine surveillance testing, and are applicable only to patients deemed appropriate candidates for surveillance testing by their treating physicians. Our sample size had limited power despite including adenocarcinoma of esophageal, GEJ, and gastric primaries. Recent work by The Cancer Genome Atlas Research Network demonstrated that genomic features of esophageal adenocarcinomas strongly resembled those of gastric adenocarcinoma, suggesting that these cancers could be considered a single disease entity.^31^ Although grouping these primaries together can increase statistical power overall, potential differences with respect to benefit of surveillance testing among these subgroups may exist and could not be evaluated in this study. Multicentre collaboration and prospective data will likely help better understand additional questions such as which clinicopathological features associate with successful salvage and palliative systemic therapy. The lack of a standardized surveillance protocol may result in variations of surveillance testing and suboptimal capture of recurrence, and whether a uniform surveillance program would lead to different outcomes. However, the vast majority of patients received CT scan every 3‐6 months, and most of the variation were seen with surveillance endoscopies and tumor markers. This reflects the uncertainty among care providers regarding the optimal surveillance protocol in this disease, and lack of high level evidence. It is unknown which components of surveillance strategy can help improve outcomes without a comparative surveillance strategy. These data represent contemporary surveillance patterns and outcomes at a high volume academic centre. Although we could not accurately account for lead time bias without detailed modeling of tumor burden at recurrence, as discussed above, lead time likely accounted for only a small component of PRS.

## 5 CONCLUSIONS

In the absence of randomized controlled trials, our results call into question routine surveillance in all patients with resected gastroesophageal adenocarcinoma. Most recurrences were distant and successful salvage therapy was rare. Routine surveillance did not seem to detect recurrences earlier or extend duration of palliative chemotherapy for the majority of patients. Future prospective clinical trials are warranted to elucidate the most optimal surveillance strategy in this disease.

## CONFLICT OF INTEREST

CH Lim has received Honorarium from Ipsen. RW Jang has a consulting or advisory role for Ipsen and Novartis; research funding from AstraZeneca, Merck, Novartis, Lilly, Boston Biomedical, and BMS. Other authors declare no conflict of interest.

## AUTHOR CONTRIBUTION

Study concepts: DM Jiang, RW Jang, and E Elimova; Study design: DM Jiang, RW Jang, and E Elimova; Data acquisition: DM Jiang, C Suzuki, CH Lim, LX Ma, P Sun, HW Sim, A Natori, BA Chan, and S Moignard; Quality control of data: DM Jiang, C Suzuki; Data analysis and interpretation: O Espin‐Garcia and DM Jiang; Manuscript preparation: DM Jiang; Manuscript review: all authors.

## Supporting information

>  Click here for additional data file.

## Data Availability

De‐identified data without patient health information may be shared upon request and approval by the corresponding author. Data‐sharing agreement is required.
