# Supplementary material for: Surveillance and outcomes after curative resection for gastroesophageal adenocarcinoma
Source: Cancer Med. 2020 Mar 4;9(9):3023–32. doi: 10.1002/cam4.2948 (PMC7196047; doi:10.1002/cam4.2948)
Supplement: Supplementary file 1 — > [file CAM4-9-3023-s001.docx]

**Electronic Supplementary Material**

**Title:**

Surveillance and Outcomes After Curative Resection for Gastroesophageal Adenocarcinoma

**Authors**:

Di. M. Jiang^a^, Chihiro Suzuki^a^, Osvaldo Espin-Garcia^b^, Charles H. Lim^a^, Lucy X. Ma^a^, Peiran Sun^a^, Hao-Wen Sim^a^, Akina Natori^a^, Bryan A. Chan^a^, Stephanie Moignard^a^, Eric X. Chen^a^, Geoffrey Liu^a^, Carol J. Swallow^c^, Gail E. Darling^d^, Rebecca Wong^e^, Raymond W. Jang^a^, Elena Elimova^a^

**Corresponding Author:**

Dr. Elena Elimova MD, MSc, FRCPC

Division of Medical Oncology, Department of Medicine

Princess Margaret Cancer Center, University Health Network, University of Toronto

610 University Ave, Toronto, Canada, M5G 2C1

Tel. 1 (416) 946-4501 x 2520

Fax. 1 (416) 946-6546

[Elena.elimova@uhn.ca](mailto:Elena.elimova@uhn.ca)

Supplementary 1. Patient selection and inclusion flow diagram

Locally advanced gastric, gastroesophageal junction, or esophageal carcinoma

(n=504)

Not assessed for eligibility

- No curative surgery (n=36)
- Squamous cell carcinoma without adenocarcinoma, or cervical esophageal cancer (n=80)

Excluded

- Metastatic disease or death within 30 days of curative surgery (n=87)
- Follow up elsewhere (n=91)

Resected locally advanced gastric, gastroesophageal junction, or esophageal adenocarcinoma

(n=388)

Resected locally advanced gastric, gastroesophageal junction, or esophageal adenocarcinoma, surveyed at PMCC

(n=210)

Supplementary 2. Surveillance patterns.

| **Surveillance specialties** | ***n* (%)** |
| --- | --- |
| MO only | 10 (5) |
| RO only | 6 (3) |
| SO only | 56 (27) |
| RO + MO | 18 (9) |
| SO + MO | 54 (26) |
| SO + RO | 19 (9) |
| SO + RO + MO | 47 (22) |

| **Surveillance testing modality** | ***n* (%)** |
| --- | --- |
| Imaging only | 142 (68) |
| Imaging + EGD | 36 (17) |
| Imaging + EGD + TM^a^ | 5 (2) |
| Imaging + TM^a^ | 7 (3) |
| TM^a^ | 2 (1) |
| None (clinical visits only) | 18 (9) |

^a^CA19-9, CEA, or CA125

Supplementary 3. Secondary malignancies discovered during surveillance.

| **Secondary malignancy** | **Stage** | **Treatment** |
| --- | --- | --- |
| Melanoma | Metastatic | Palliative |
| Melanoma | Metastatic | Palliative |
| Colorectal | Metastatic | Palliative |
| Colorectal | Local | Curative resection and adjuvant chemotherapy |
| Pancreatic | Metastatic | Palliative |
| Lung (squamous cell carcinoma) ^a^ | N2 | Definitive chemoradiation, later recurred with metastatic disease |

^a^had surgical resection alone for a T1aN0M0 gastric cancer.

Supplementary 4. Outcomes and characteristics of patients who underwent an intensive surveillance strategy (surveillance imaging intervals $\leq$4 months), versus other patients surveyed (surveillance imaging interval >4 months). The arbitrary threshold of 4 months was chosen based on previous randomized trials in colorectal cancer [Grossmann *et al*. Surg. Oncol. 2004; 13:119–124. Renehan *et al*. BMJ 2002; 324:1–8. Pita-Fernandez *et al*. Ann. Oncol. 2015; 26:644–656.]

|  | Intensive Surveillance *n*=67 (%) | Nonintensive Surveillance *n*=123 (%) | *p* value |
| --- | --- | --- | --- |
| 5-year OS rate | 13% | 18% | <0.001 |
| 5-year DFS rate | 6% | 72% | <0.001 |
| Median TTR^a^ | 14.5 months | 24.0 months | <0.001 |
| Median PRS^a^ | 11.2 months | 16.3 months | 0.33 |
| Gastric primaries | 31% | 51% | 0.029 |
| Positive margin | 15% | 4% | 0.049 |
| High risk pathologic staging | 79% | 65% | 0.048 |

^a^patients who relapsed

Supplementary 5. Characteristics of patients who received post recurrence therapy.

|  | Surveillance-detected recurrence  *n* (%) | Symptomatic recurrence  *n* (%) |
| --- | --- | --- |
| Attempted Salvage | 15 (7.1) | 1 (0.5) |
| Successful | 4 (1.9) | 0 |
| Unsuccessful | 6 (2.9) | 0 |
| Immature follow up | 5 (2.4) | 1 (0.5) |
| Palliative chemotherapy | 28 (13.3) | 18 (8.6) |
| Median duration | 3.9 mo (range 0^a^-26.0) | 3.3 mo (range 0^a^-14.6) |
| Number of lines  1  2  3 | 18 (64)  8 (29)  2 (7) | 12 (67)  6 (33)  0 |

^a^only received 1 cycle of chemotherapy

Supplementary 6. Characteristics of patients who received successful salvage Therapy

| **Primary** | **Primary treatment** | **Recurrence** | **Salvage tx** |
| --- | --- | --- | --- |
| Esophagus | Surgery alone | supraclavicular mediastinal lymph node | Chemoradiation |
| AEG2 | Preoperative chemoradiation and surgery | Small bowel | Small bowel resection plus adjuvant chemotherapy |
| AEG2 | Surgery alone | RUL^a^ | RUL lobectomy |
| Gastric | Surgery alone | Gastrohepatic ligament lymph node | Neoadjuvant chemotherapy and total gastrectomy |

^a^RUL, right upper lobe
